# Structural Characteristics, Rheological Properties, and Antioxidant and Anti-Glycosylation Activities of Pectin Polysaccharides from Arabica Coffee Husks

**DOI:** 10.3390/foods12020423

**Published:** 2023-01-16

**Authors:** Zelin Li, Bin Zhou, Tingting Zheng, Chunyan Zhao, Yan Gao, Wenjun Wu, Yingrun Fan, Xuefeng Wang, Minghua Qiu, Jiangping Fan

**Affiliations:** 1 College of Food Science and Technology, Yunnan Agricultural University, Kunming 650201, China; 2 State Key Laboratory of Phytochemistry and Plant Resources in West China, Kunming Institute of Botany, Chinese Academy of Sciences, Kunming 650201, China

**Keywords:** Arabica coffee, pectin polysaccharide, structure, rheological property, anti-glycosylation, molecular docking

## Abstract

As primary coffee by-products, Arabica coffee husks are largely discarded during coffee-drying, posing a serious environmental threat. However, coffee husks could be used as potential material for extracting pectin polysaccharides, with high bioactivities and excellent processing properties. Thus, the present study aimed to extract the pectin polysaccharide from Arabica coffee husk(s) (CHP). The CHP yield was calculated after vacuum freeze-drying, and its average molecular weight (Mw) was detected by gel permeation chromatography (GPC). The structural characteristics of CHP were determined by Fourier transform infrared spectroscopy (FT-IR), X-ray diffraction (XRD), proton nuclear magnetic resonance (^1^H NMR), and scanning electron microscopy (SEM). Additionally, the rheological and antioxidant properties of CHP and the inhibition capacities of advanced glycation end products (AGEs) with different concentrations were evaluated. The interaction mechanisms between galacturonic acid (GalA) and the AGE receptor were analyzed using molecular docking. The results demonstrated that the CHP yield was 19.13 ± 0.85%, and its Mw was 1.04 × 10^6^ Da. The results of the structural characteristics results revealed that CHP was an amorphous and low-methoxyl pectic polysaccharide linked with an α-(1→6) glycosidic bond, and mainly composed of rhamnose (Rha, 2.55%), galacturonic acid (GalA, 45.01%), β-N-acetyl glucosamine (GlcNAc, 5.17%), glucose (Glc, 32.29%), galactose (Gal, 6.80%), xylose (Xyl, 0.76%), and arabinose (Ara, 7.42%). The surface microstructure of CHP was rough with cracks, and its aqueous belonged to non-Newtonian fluid with a higher elastic modulus (G′). Furthermore, the results of the antioxidant properties indicated that CHP possessed vigorous antioxidant activities in a dose manner, and the inhibition capacities of AGEs reached their highest of 66.0 ± 0.35% at 1.5 mg/mL of CHP. The molecular docking prediction demonstrated that GalA had a good affinity toward AGE receptors by −6.20 kcal/mol of binding energy. Overall, the study results provide a theoretical basis for broadening the application of CHP in the food industry.

## 1. Introduction

Coffee, one of the most popular beverages in the world, is an essential agricultural product in the international trade market [1]. In recent years, China has recorded a significant proliferation in coffee consumption, which is expected to become the most crucial global coffee trade and consumption market [2], with a total coffee cherry yield of 350,000 tons and more than CNY 2.58 billion in output value annually [3]. Meanwhile, the coffee by-product production will increase with increased coffee bean processing, such as coffee husks, accounting for about 45–50% of the coffee cherry and an annual yield of about 160,000 tons [4]. However, a large number of coffee by-products will lead to soil structure damage and environmental pollution upon inadequate disposal. So far, only a tiny part of coffee husks has been used to process biofuel, feed, biosorbents, tea, and enzymes [5]. Nevertheless, coffee husks could serve as potential resources for obtaining pectin [6].

Generally, pectin-type polysaccharides containing galacturonic acid (GalA) linked at the α-1,4 positions are responsible for the rigid texture of fruits [7]. These polysaccharides could be applied in food manufacturing due to their sound processing performances (such as in jams, drinks, ice creams, bread, etc.) and antioxidant and anti-glycosylation activities [8]. For instance, pectin polysaccharides isolated from some fruit peels could inhibit the formation of advanced glycation end products (AGEs) produced by non-enzymatic glycation [9]. AGEs, a major indicator of glycosylation, is a complex, irreversible product [10]. Excessive AGEs in the body could lead to chronic diseases, such as diabetes, kidney disease (uremia), stroke, Alzheimer’s disease, and accelerated aging [11]. Unfortunately, very few studies have been reported so far on the structural and rheological properties of pectin polysaccharide from Arabica coffee husk and its anti-AGE activities. For instance, Hasanah et al. [12] extracted the pectin from coffee husks Arabica Gayo and detected the functional group and microstructure by FT-IR and SEM methods. However, they did not analyze the monosaccharide composition and bioactivity of pectin. Reichembach et al. [4] evaluated the gelling properties of pectin from coffee (*Coffea arabica* L.) pulp.

Studies have reported that different structures, rheological properties, and antioxidant activities of pectin polysaccharides could affect their processing properties. For example, a pectin-type polysaccharide from galangal with a lower Mw and more of a rhamnogalacturonan I (RG I) structure could be processed as an efficient emulsifier to construct the flavonoid delivery system [13]. Therefore, this study aimed to obtain purified CHP and characterize its structure. Additionally, the rheological properties, antioxidant activity, and AGE inhibitory activities in the bovine serum albumin (BSA)-glucose system were evaluated.

## 2. Materials and Methods

### 2.1. Materials and CHP Preparation

The dried Coffea arabica L. husks were obtained from Baoshan Beaton Coffee Co., Ltd., in Yunnan Province, China. The husks’ moisture was less than 5%. The dried husks were ground and sieved through a 60 mesh ASTM E11 stainless steel sieve (Endecotts Co., Ltd., London, UK) to obtain the coffee husk powder.

The pectin polysaccharides were extracted according to the methods reported in our previous study [14]. Briefly, 500 g of coffee husk powder was accurately weighed and extracted using 1.5% cellulase at a solvent pH of 4.9, a solid–liquid ratio of 1:24.65 (g/mL), and an extraction time of 3 h at 45 °C. The enzyme was removed using boiling water for 10 min. The extract was filtered using a polyester fabric and centrifuged at 4000× *g* for 15 min. Then, the extract was precipitated using 4 volumes of absolute ethanol and stored overnight at 4 °C. The precipitate pectin was gathered by centrifugation at 10,000× *g* for 20 min, washed thrice with ethanol, and vacuum freeze-dried. Further, 100 mL of 1.5 mg/mL crude pectin polysaccharide solution was prepared with distilled water. The impurities of crude pectin (such as pigment and protein) significantly affect its purity and physicochemical characteristics, so it needs decolorization and deproteinization treatment [15]. Accurately, 7 g of AB-8 macroporous resin was mixed with the crude pectin polysaccharide solution at 55 °C, 130 revolutions per minute (r/min) for oscillating de-coloration for 5 h [14]. Then, the solution was concentrated to half of the original volume at 50 °C and four times the volume of the Sevage solution (chloroform: n-butanol = 4:1, *v/v*) was re-added to deproteinize [16]. After deproteinization and de-coloration, the solution was dialyzed under tap water and distilled water for 24 h, respectively, and vacuum freeze-dried to obtain the CHP.

### 2.2. Physicochemical Indexes

The moisture and ash contents were detected according to the method reported by Lal et al. [17]. The total amount of carbohydrates was determined by the phenol–sulfuric acid method [18] using a standard glucose curve (with an R^2^ of 0.9945) at 490 nm. The polyphenol content was determined by using the Folin–Ciocalteu method [19], and the standard curve of gallic acid was obtained with an R^2^ of 0.9951. The flavone content was detected using the ultraviolet and visible spectrophotometry methods according to the previously reported method of Capek et al. [20], and the standard curve of rutin was obtained at an R^2^ of 0.9936. Polyphenol and flavone were defined as mg gallic acid and rutin equivalent per kilogram of CHP. The degree of esterification (DE) and degree of acetylation (DA) were evaluated using the titrimetric method as demonstrated by Song et al. [21] and calculated using the following Formulas (1) and (2):

(1) $$\mathrm{DE}=\frac{V_{2}}{V_{1}+V_{2}}\times 100\%$$

where *V*_1_ represents the consumed volume of the first titrated NaOH; *V*_2_ represents the consumed volume of the second titrated NaOH.

(2) $$\mathrm{DA}=\frac{V_{3}}{V_{1}+V_{2}+V_{3}-V_{4}}\times 100\%$$

where *V*_1_ represents the consumed volume of NaOH of the initial titration stage; *V*_2_ represents the consumed volume of NaOH of the saponification titration stage; *V*_3_ represents the NaOH consumed volume of the amide titration stage; *V*_4_ represents the NaOH consumed volume of the acetic acid titration stage.

### 2.3. Molecular Weight (Mw) Detection

The Mw of the CHP sample was detected by gel permeation chromatography (GPC) using the ELEOS System instrument equipped with Shodex OHpak SERIES SB-806 tandem 803 columns and Laser (LS) and refractive index (RI) detector (Waters Co., Ltd., Milford, MA, USA). The mobile phase consisted of 0.02% (*w*/*w*) NaN_3_ at a temperature of 40 °C, and the flow rate was 1 mL/min. The Mw was calculated according to the calibration curve of standard dextran.

### 2.4. Detection of Monosaccharide Composition

The monosaccharide composition of CHP was determined using the high-performance liquid chromatography (HPLC) method [15]. The sample was dissolved in distilled water and hydrolyzed by 2 mol/L trifluoroacetic acid (TFA) solution at 100 °C for 12 h. For the impurities removal, the solution was immediately cooled and centrifuged at 4000× *g* condition for 10 min. The liquid phase was then freeze-dried to remove the trifluoroacetic acid. After dissolving in deionized water, the hydrolysates were injected into an HPLC system (Agilent, Nara, Japan) equipped with an Agilent, ZORBAX SB-C18 (4.6 × 150 mm, 5 μm) column and VWD ultraviolet detector (Agilent, Nara, Japan). The mobile phases A and B consisted of 15% acetonitrile + 85% KH_2_PO_4_-NaOH buffer (pH 6.9) and 40% acetonitrile + 60% KH_2_PO_4_-NaOH buffer (pH 6.9) with an elution gradient of 0–25 min. The flow rate was 1 mL/min. The monosaccharide composition was determined according to the retention time of the standard monosaccharides: rhamnose (Rha), arabinose (Ara), mannose (Man), fucose (Fuc), galactose (Gal), galacturonic acid (GalA), glucuronic acid (GlcA), ribose (Rib), glucose (Glc), xylose (Xyl), glucosamine (GlcN), and β-N-acetyl glucosamine (GlcNAc).

### 2.5. X-ray Diffraction (XRD) Detection

The XRD analysis of the CHP was performed using an Ultima IV X-ray diffractometer (Beijing crown far science and technology Co., Ltd., Beijing, China) at a diffraction angle (2θ) from 5° to 60°, a voltage of 40 kV, and a current of 40 mA. The 2θ increment was 2° with a counting time of 1 s/step.

### 2.6. Fourier Infrared Spectroscopy (FT-IR) Detection

The CHP was ground with spectral potassium bromide (KBr) powder in an agate mortar and then pressed into 1 mm thin slices for Bruker Tensor 27 FT-IR (Bruker Daltonics Co., Ltd., Karlsruhe, Germany) spectrometer analysis within 4000–500 cm^−1^ at a resolution of 4 cm^−3^. The test mode was set to the powder conventional tablet pressing mode.

### 2.7. Proton Nuclear Magnetic Resonance (^1^H NMR) Detection

A total of 20 mg of CHP was accurately weighed and dissolved in 1 mL of D_2_O (purity: 99.8%). It was stirred at room temperature overnight and detected by the Bruker 134 AV600 mass spectrometer (Bruker Daltonics Co., Ltd., Karlsruhe, Germany) at 25 °C.

### 2.8. Scanning Electron Microscopy (SEM) Detection

A freeze-dried CHP sample was coated with a thin gold layer and photographed using a scanning electron microscope (Tescan Mira4, Brno, Czech Republic) at an accelerating voltage of 30 kV. The SEM images were recorded at 200×, 500×, 1000×, and 2000× magnification.

### 2.9. Detection of Rheological Properties

The rheological characteristics of CHP samples (2.5 mg/mL) were investigated using a DHR-2 controlled strain rheometer (TA Instruments Co., Ltd., Newcastle, DE, USA) equipped with a cone plane geometry (40 mm diameter, 1000 μm clearance) according to the previously reported methods [22]. The steady shear test was employed from 0.1 to 1000 s^−1^ at 25 °C, and the frequency scanning test was performed within the range from 0.1 to 10 rad·s^−1^ at a strain of 2% (within the linear viscoelastic region. The elastic modulus (G′, Pa) and viscous modulus (G″, Pa) were recorded at 25 °C.

### 2.10. Antioxidant Activity Assay

#### 2.10.1. DPPH and Hydroxyl (-OH) Radical Scavenging

The DPPH and hydroxyl radical scavenging assay were conducted based on the methods reported by Mzoughi et al. [23]. Briefly, 150 microliters of CHP solutions (0.1–10 mg/mL) were mixed with 50 μL of 0.2 mmol/L DPPH solution. After reacting for 30 min in the dark environment, the absorbance was recorded at 517 nm. As for the -OH, 1 mL of 10 mmol/L salicylic acid and distilled water were added to 1 mL of 10 mmol/L FeSO_4_. Then, 1 mL of CHP and 8.8 mmol/L of H_2_O_2_ were added to the solution pool. The absorbance was measured at 510 nm after incubating for 30 min at 37, and the scavenging rate was calculated using the following Formula (3):

(3) $$\mathrm{Scavenging}\;\mathrm{rate}=\left(1-\frac{A_{2}-A_{3}}{A_{1}}\right)\times 100\%$$

where *A*_1_, *A*_2_, and *A*_3_ are the absorbances of the blank control, sample, and background group, respectively.

#### 2.10.2. ABTS^+^ Radical Scavenging

The ABTS^+^ radical scavenging assays were performed according to the methods reported by Song et al. [24]. A total of 88 microliters of potassium persulfate solution (140 mmol/L) was mixed with 5 mL of ABTS^+^ solution (7 mmol/L) and incubated in the dark for 24 h. After diluting 50 times, the absorbance of the working solution was standardized to 0.7 ± 0.02 at 734 nm using pH 4.5 acetate buffer. Later, 200 μL of each CHP solution and working solution were immediately mixed to measure the absorbance at 734 nm, and the scavenging rate was calculated using the following Formula (4):

(4) $$\mathrm{Scavenging}\;\mathrm{rate}=\left(1-\frac{A_{2}}{A_{1}}\right)\times 100\%$$

where *A*_1_ and *A*_2_ are the absorbances of the control and sample, respectively.

#### 2.10.3. Superoxide Radical (O_2_^−^) scavenging 

The superoxide radical (O_2_^−^) scavenging assay was performed according to the method described by Chen et al. [25]. Accurately, 4.5 mL of Tris-HCl (0.05 mol/L, pH 8.2) was mixed with 1 mL of ultrapure water at 25 °C for 20 min. Then, 1 mL of CHP and 5 mL of pyrogallol were immediately mixed at 25 °C for 5 min. Further, 1 mL of HCl was immediately added and mixed to measure the absorbance at 320 nm, and the scavenging capacity was calculated using the following Formula (5):

(5) $$\mathrm{Scavenging}\;\mathrm{rate}=\left(1-\frac{A_{3}-A_{2}}{A_{1}}\right)\times 100\%$$

where *A*_1_, *A*_2_, and *A*_3_ are the absorbance of the control, background, and sample group, respectively.

Vitamin C (Vc) with the same concentration gradient was used as a positive control in all radical scavenging tests.

### 2.11. Anti-Glycosylation Activity Assay

The non-enzymatic glycosylation system of bovine serum albumin (BSA)-glucose was constructed based on the methods reported by Amamou et al. [26]. Accurately, 10 mL of bovine serum albumin (BSA, 20 mg/mL), 5 mL of glucose solution (500 mmol/L), and 5 mL of phosphate buffer (200 mmol/L, pH 7.4) containing 0.02% (*w*/*w*) of sodium azide were mixed. This mixture was considered a negative control group and then mixed with 5 mL of different CHP solutions (0.5, 1.0, 1.5 mg/mL) to obtain the reaction system. The same treatment of aminoguanidine (AG) was considered the positive control group. After incubation for 0, 7, 14, 21, and 28 days at 37 ± 0.5 °C, 0.5 mL was taken out of the above reaction solution and diluted to 10 mL with phosphate buffer (200 mmol/L, pH 7.4), respectively. The fluorescence intensities of AGEs were detected by the Varioskan LUX fluorescence microplate reader (Thermo Fisher Scientific Co., Ltd., Waltham, MA, USA). The excitation wavelength was set at 370 nm, and the emission wavelength was set at 440 nm. The inhibition rate of AGEs was calculated using the following Formula (6):

(6) $$\mathrm{Inhibition}\;\mathrm{rate}=\frac{A_{0}-A_{1}}{A_{0}}\times 100\%$$

where *A*_0_ and *A*_1_ represent the absorbance of the negative control sample and the positive control sample or CHP sample, respectively.

### 2.12. Molecular Docking between GalA and AGE Receptor

The total binding energy for the interaction between the GalA and AGE receptors was determined based on the energy contributed by the hydrogen bonding, van der Waals, and electrostatic interaction [27]. Herein, the 3D conformer of AGE receptors (PDB ID: 6XQ1) and GalA (compound ID: 439215) was downloaded from the RCSB (https://www.rcsb.org, accessed on 25 April 2022) and PubChem database (http://pubchem.ncbi.nlm.nih.gov, accessed on 25 April 2022), respectively. AutoDock was applied for molecular docking simulation, and the grid coordinates of the binding box of the AGE receptors were identified as center x: −34.86, center y: −11.52, and center z: 3.90 with dimensions size x: 70, size y: 40, and size z: 126. The semi-flexible docking was employed: the protein structure was kept rigid, and all the torsional bonds of GalA were set free. The optimal binding position of the complex was the model with the lowest binding energy. PyMOL and LigPlot were conducted to visualize the interactions between the GalA and AGE receptors [28].

### 2.13. Statistical Analysis

All experiments were performed in triplicate, and the data were represented as mean ± standard deviation (SD). The figures were created by the Origin 2021 software (OriginLab Co., Ltd., Hampton, MA, USA). The significant differences were determined by Duncan analysis using the SPSS 18.0 software (International Business Machines Co., Ltd., Armonk, NY, USA), and different letters represent the significant differences (*p* < 0.05).

## 3. Results and Discussion

### 3.1. Physicochemical Properties

As shown in Table 1, the CHP yield from 100 g of dried crude pectin polysaccharide was 19.13 ± 0.85%. The moisture and ash of CHP were 5.08 ± 0.23% and 4.81 ± 0.33%, respectively. The ash content was lower than the food-grade (FAO recommended lower 10%) pectin [17], suggesting that CHP has higher quality. Moreover, the contents of carbohydrates, flavonoids, and polyphenols of CHP were 85.58 ± 1.02%, 1.39 ± 0.13 mg/kg, and 6.93 ± 0.10 mg/kg, respectively. Compared with other pectin isolated from coffee pulp Arabica Gayo [12], CHP yield was higher, which might be attributed to the different extraction conditions of CHP.

**Figure 1:**
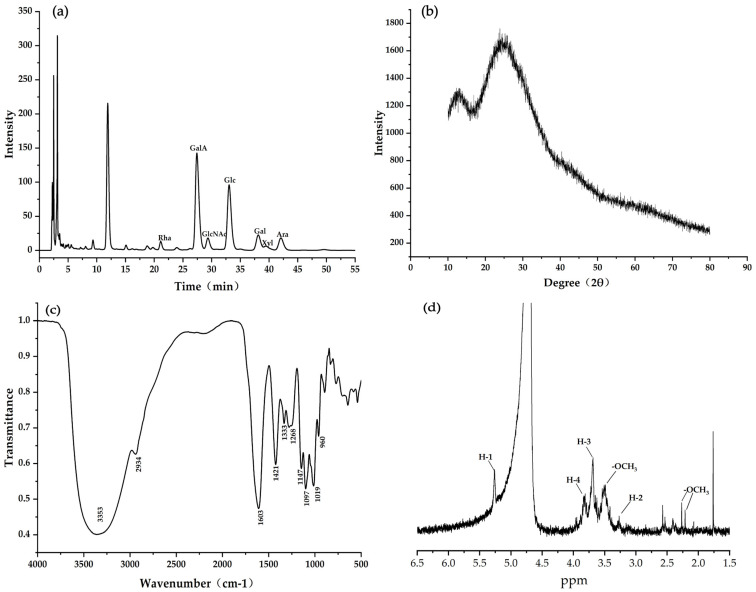
Structure and composition of CHP. (**a**) Monosaccharide composition; (**b**) XRD; (**c**) FT-IR; (**d**) ^1^H NMR.

The structure, monosaccharides composition, and GalA of pectin polysaccharides significantly differ in different pectin polysaccharides from different sources [29]. As shown in Table 1 and Figure 1a, GalA and Glc were the major monosaccharides of the CHP sample, accounting for 77.30% of the total monosaccharides. It is reported that GalA constitutes the central part of the homogalacturonan backbone (HG) and rhamnogalacturonan I and II (RG I and II) regions, contributing to its smooth nature [30]. The HG regions and RG I regions accounted for 42.46% and 19.32% of the total CHP structure, indicating that the CHP structure was smooth. Moreover, Ara, Gal, Glc, and Rha were the primary neutral sugars of CHP, and their concentration reached around 81% of all neutral sugars. These results indicated that the structure of CHP was formed by wide branches of Ara, Gal, Glc, and Rha. Additionally, the molar ratios of R_1_, R_2_, and R_3_ could contribute to the linearity of the structure. Studies have reported higher R_1_ value, more linear regions, and high RG I amount [30]. In the present study, the obtained R_1_ value (2.68) in the CHP sample was higher, while its R_2_ (0.06) and R_3_ (5.58) values were lower than some fruit peel pectin, suggesting that CHP had more linear regions with shorter side chains.

The DE and DA values are the important indexes for the functional properties and applications of pectin, and a DE value lower than 50% could be recognized as low-methoxyl pectin [31]. It is reported that low-methoxyl pectin containing many HG and RG I regions could be used to formulate low- or no-sugar products in the food industry [32]. The DE and DA values of CHP were 38.81 ± 0.96% and 16.36 ± 0.75%, respectively, suggesting that CHP had a lower methoxyl group and hence could be a potential food ingredient, such as low- or no-sugar products. The Mw of the pectin polysaccharide and its distribution showed a critical role in the bioactive properties, especially low Mw could provide better antioxidant activity [33]. The CHP showed an extensive distribution with a low Mw of 1.04 × 10^6^ Da, and its antioxidant capacity will also be further evaluated below.

### 3.2. XRD Analysis

The XRD analysis could determine the crystallinity or non-crystallinity structural properties of pectin polysaccharides. As depicted in Figure 1b, the sharp and robust absorption peaks were not detected at 10° to 80°, suggesting that the CHP was an amorphous pectin polysaccharide. This might have happened due to the difference in pectin’s dehydration and drying process and whether the partially acetylated pectin was dissolved in alcohol [34]. These results were consistent with other fruit peel pectin polysaccharides, with absorption peaks ranging from 5° to 90° [35].

### 3.3. FT-IR Analysis

The functional group signals of CHP were detected by FT-IR spectroscopy at 500–4000 cm^−1^. The signals at 3300–3500 cm^−1^ resulted from the O-H stretching; the broad signals at 2800–3000 cm^−1^ demonstrated the C-H stretching of CH, CH_2_, and CH_3_ groups [34]. As depicted in Figure 1c, the absorption bands at 3353 cm^−1^ and 2934 cm^−1^ were the stretching vibrations of the O-H and C-H bonds in the CHP sugar ring. The absorption peak at 1400–1650 cm^−1^ originated from C=O stretching vibrations of free carboxyl (COO-) and uronic acid [18]. The absorption peak at 1421 cm^−1^ and 1603 cm^−1^ indicated that CHP contained several units of GalA. Moreover, the bands between 800 cm^−1^ and 1200 cm^−1^ correspond to the fingerprint region for carbohydrates with β-type or α-(1→6) glycosidic bonds [36]. The peak observed at 960 cm^−1^, 1019 cm^−1^, 1097 cm^−1^, and 1147 cm^−1^ indicated that the CHP had β-type or α-(1→6) glycosidic bonds.

### 3.4. ^1^H NMR Analysis

As depicted in Figure 1d, the characteristic sharp signals at 2.0–3.65 ppm were assigned to the methoxyl groups of GalA units and the acetyl groups (-COCH_3_) [21,37]. The signals detected at 2.19 ppm, 2.25 ppm, 3.60 ppm, and 3.65 ppm indicated that CHP contained the -COCH_3_ and GalA group, which was consistent with the FT-IR results [38]. The anomeric hydrogen characteristic proton peaks of β-type glycoside were less than 5.0 ppm, while the α-type glycoside was contrary [39]. The characteristic proton peak (H-1) was tested at 5.50 ppm, indicating that CHP contained the α-type glycoside bond. Meanwhile, the chemical shifts of H-2, H-3, and H-4 were observed at 3.60 ppm, 3.80 ppm, and 3.90 ppm, respectively. These major anomeric proton signals ranging from 3.2–4.0 ppm confirmed the chemical shifts of the sugar ring [24].

### 3.5. Microstructure Analysis

**Figure 2:**
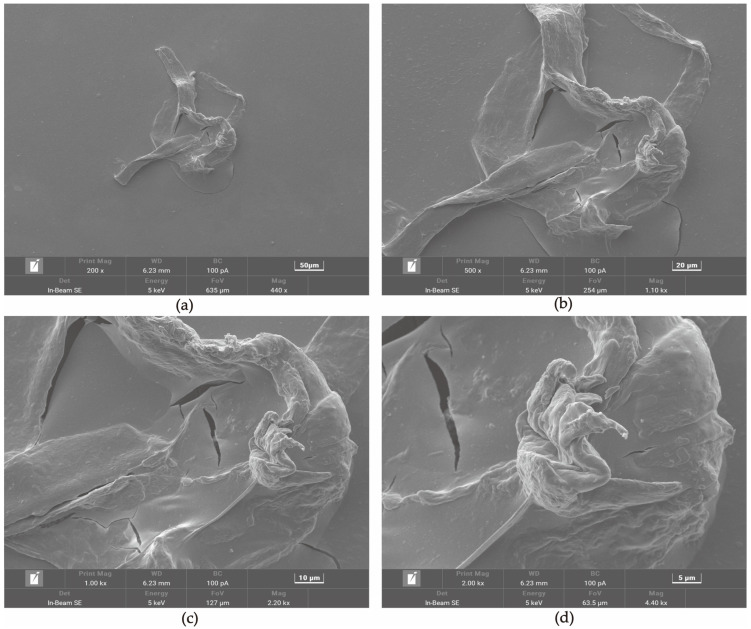
Microstructure of CHP at different magnifications. The sample was magnified by 200, 500, 1000, and 2000 times, (**a**–**d**).

Distinctive surface microstructure of freeze-dried CHP was observed by SEM at different magnifications from 200× to 2000× (Figure 2a–d). The CHP first observation was found to be rough, dense, and compact, with small particles adhering to the surface. A ruptured surface in the middle was observed as a ripped-apart structure. Furthermore, the surface appeared as a wrinkled-like surface around the ruptured one, which might have happened due to the energy and gas released by enzymes and heating during the extraction process [40]. Additionally, the dense surface of pectin polysaccharides showed better structural properties, which could precisely bind with the active compounds, and the degrees of branching could influence its surface morphology and disturb the bonding effect [7]. However, the different extraction methods and variety significantly contributed to the microstructural difference. The pectin from coffee pulp Arabica Gayo showed a smooth area and homogenous surface when extracted with different concentrations of citric acid [12], which was contradictory to CHP by cellulase extraction. Pectin polysaccharide from Kiwano peels showed an irregular block with some circular cavity surface [7], which was not observed in the CHP.

### 3.6. Rheological Characteristics Analysis 

**Figure 3:**
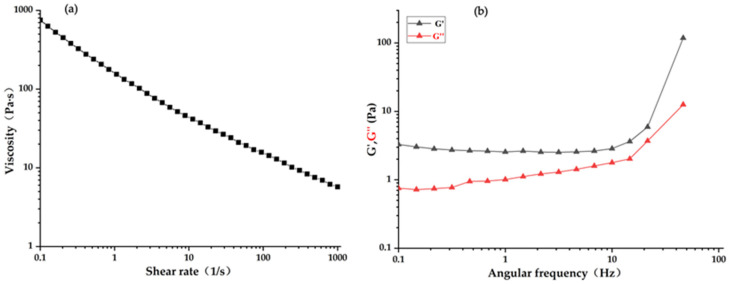
Rheological behaviors of CHP. (**a**) Relationship of apparent viscosity on shear rate; (**b**) elastic modulus (G′) and viscous modulus (G″) against angular frequency.

The apparent viscosity of pectin polysaccharide appears as a shear-thinning phenomenon with the increase in shear rate, and its aqueous solution belongs to the non-Newtonian fluid [41]. As depicted in Figure 3a, the apparent viscosity of CHP significantly decreased with the increase of shear rate from 0.1 to 1000 s^−1^, indicating that it possessed non-Newtonian shear-thinning flow behaviors in aqueous solutions. This might be attributed to the breakdown of pectin polysaccharide molecules’ rearrangement and their physical interactions under shear acceleration [42]. Furthermore, the G′ and G″ could well evaluate the pectin polysaccharide’s application, processing, and stabilization properties, but the number and length of side chains, molecular weight, and demethylation reaction significantly affect these properties [43]. In the range of 0–99 Hz, the G′ of the CHP sample was larger than that of the G″ (Figure 3b), implying that the CHP sample had good elastic characteristics. Pectin polysaccharide has good elastic characteristics and a structural network to bind and stabilize other ingredients [44]. The low-methoxyl CHP could, thus, be applied to the food industry as a stabilizer and delivery of material.

### 3.7. Antioxidant Activity of CHP

**Figure 4:**
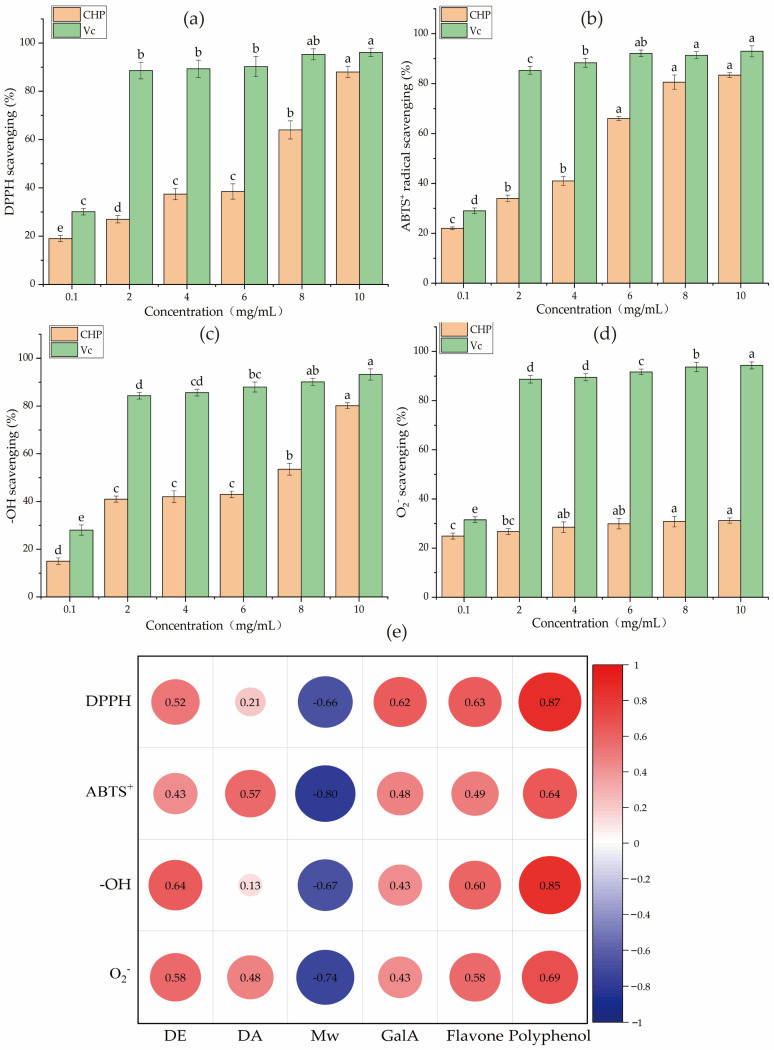
The antioxidant capacity of CHP; (**a**–**d**) represent the scavenging ability of CHP to DPPH, ABTS^+^, hydroxyl radical, and superoxide radical, respectively, different letters on the bars represent the significant differences (*p* < 0.05); (**e**) represents the correlation among DE, DA, Mw, GalA, polyphenols, flavonoids, DPPH, ABTS^+^, hydroxyl radical, and superoxide radical scavenging capacities.

The antioxidant activities of CHP were evaluated by four kinds of scavenging radical assays. As depicted in Figure 4a–d, the CHP aqueous solutions displayed antioxidant activities in a concentration-dependent manner. CHP had the lowest scavenging ability of 31.20 ± 0.05 % s on superoxide radical (O_2_^−^) at 10 mg/mL. The highest DPPH, ABTS^+^, and hydroxyl radical (-OH) scavenging rates of CHP samples at 10 mg/mL were 88.00 ± 0.37%, 83.40 ± 0.93%, and 80.20 ± 0.23%, respectively. The IC_50_ of DPPH, ABTS^+^, and -OH were 4.31 mg/mL, 2.14 mg/mL, and 4.31 mg/mL, respectively. Furthermore, the positive control Vc samples showed a similar trend up to the highest effect at 2–10 mg/mL on the DPPH, ABTS^+^, O_2_^−^, and -OH scavenging rates, respectively, but it exhibited lower IC_50_ values than CHP on four radical scavenging rates. It is reported that the antioxidant capacity of pectin polysaccharides is different from the contents of GalA, Mw, flavonoids, polyphenols, DE, and DA [45]. Siu et al. [46] showed that polysaccharides with low Mw could increase antioxidant activity. Popov et al. [47] also found that fireweed pectin polysaccharides with low Mw and high GalA content exhibit better antioxidant properties. The antioxidant capacities of pectin polysaccharides could be attributed to the contents of hydroxyl groups and electron transfer (such as ROH or RO–) from pectin to DPPH, ABTS^+^, and other radicals [48]. Therefore, Pearson correlation analysis was conducted between the above indexes and antioxidant activity (Figure 4e), and the results showed that the antioxidant activities of CHP were negatively correlated with Mw and positively correlated with GalA, flavonoids, polyphenols, DE, and DA. Of them, polyphenol showed a significantly positive correlation with DPPH scavenging with a correlation coefficient of 0.87, while the Mw showed a negative correlation with ABTS^+^ scavenging with a correlation coefficient of 0.80.

### 3.8. Anti-Glycosylation Activity of CHP

**Figure 5:**
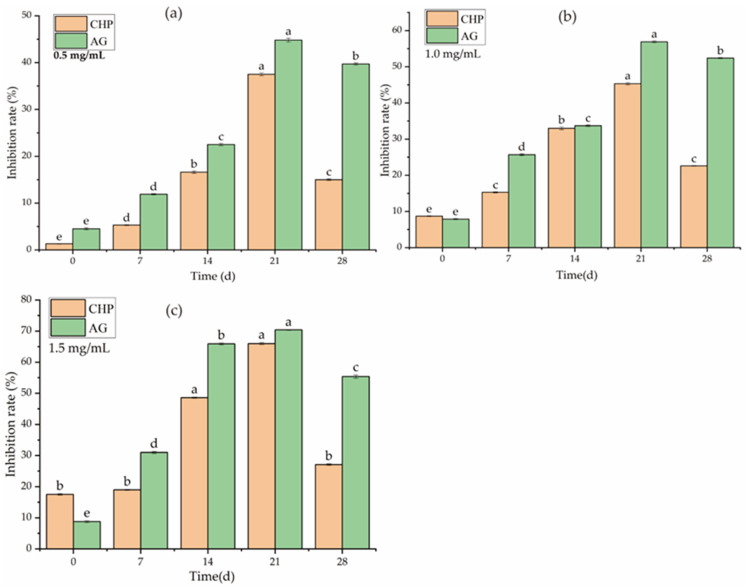
AGE inhibition capacity of CHP; (**a**–**c**) represent the inhibitory effect of CHP samples at different concentrations (0.5, 1.0, and 1.5 mg/mL) on AGE formation, different letters on the bars represent the significant differences (*p* < 0.05).

AGEs are oxidizing compounds that could be formed and accumulated in the natural environment by proteins and reducing sugars both in vivo and in vitro [26]. It is reported that pectin polysaccharides with high antioxidant activities could effectively inhibit the formation of AGEs, attributed to the high content of GalA units, which could reduce the release of reactive oxygen species [49]. As depicted in Figure 5a–c, the inhibition activities of CHP presented a dose-dependent effect at the whole incubation stage (0–28 days). After 21 days of incubation, the inhibition rates reached the maximum of 37.50 ± 0.31% (0.5 mg/mL), 45.30 ± 0.29% (1.0 mg/mL), and 66.00 ± 0.35% (1.5 mg/mL), respectively. Although the inhibition effect of the aminoguanidine (AG) solution was stronger than CHP, both inhibition effects significantly decreased (*p* < 0.05) in 28 days, probably due to substrate depletion in the reaction system. Therefore, it was speculated that the potential anti-glycosylation mechanism of CHP broke down the GalA bond and released active hydroxyl and carboxyl groups during incubation. These active groups could enter the BSA-glucose system and bind with radicals to form water or other harmless compounds [50]. Another speculation was that the GalA of CHP bound with AGE receptors in the early stage to reduce the formation of AGEs [27]. In this study, 45.01% of GalA was detected in the CHP sample (Table 1), implying that CHP has an anti-glycosylation effect by inhibiting the formation of AGEs. Additionally, high DE value, branching structures (RG I and RG II), low Mw, and pectin polysaccharide substrate concentration are the critical indicators affecting its anti-glycosylation activity [51]. The AGE inhibition rate of pectin oligosaccharide from *Actinidia arguta* fruit (1.5 mg/mL) was 52.92% after 28 days of incubation [52], which was higher than that of CHP at the same stage. This could be attributed to the pectin oligosaccharide from *Actinidia arguta* fruit containing higher GalA (83.67%) and DE (40.54%) than CHP. Zhao et al. [53] studied the AGE inhibition rates of polysaccharides from 9 species of *Polygonatum* ssp. (1.5 mg/mL), and found lower GalA (3.9–34.7%) below 30% after 21 days of incubation. This is contradictory to the GalA in CHP at the same level.

### 3.9. Molecular Docking Analysis

**Figure 6:**
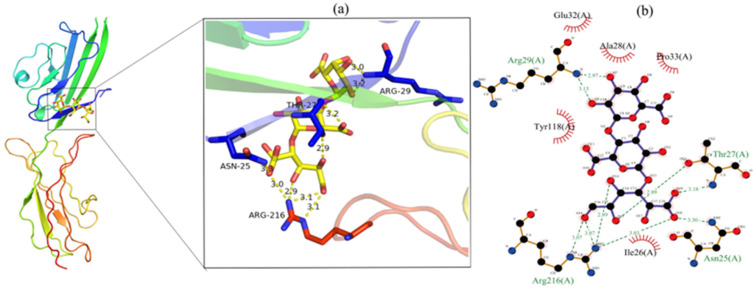
The molecular docking results; (**a**) 3D plot of the interaction between the GalA and AGE receptor; (**b**) 2D ligand interaction plot of GalA docking with the AGE receptor.

The binding affinity of the AGE receptor–ligand (GalA) complex was evaluated by molecular docking. As depicted in Figure 6, GalA interacted with amino acid residues (Arg216, Asn25, Thr27, and Arg29) to form nine hydrogen bonds and with Ile26, Pro33, Ala28, Glu32, and Tyr118 to form hydrophobic groups. It is reported that hydrogen bond formation could reduce the hydrophilicity, while increased BSA hydrophobicity could increase complex stability, thereby reducing the formation of AGEs [27]. The docking results showed a binding energy of 6.2 kcal/mol, indicating the GalA of CHP had a good binding affinity with the AGE receptor. These results suggest that GalA of CHP plays a crucial role in inhibiting AGE production.

## 4. Conclusions

The CHP was an amorphous and low-methoxyl pectin polysaccharide (DE value, 38.81 ± 0.96%) with an α-(1→6) glycosidic bond, and its monosaccharides consisted of Rha (2.55%), GalA (45.01%), GlcNAc (5.17%), Glc (32.29%), Gal (6.80%), Xyl (0.76%), and Ara (7.42%). The average Mw was 1.04 × 10^6^ Da. The microstructure of CHP demonstrated that its surface was dense and cracked. The CHP sample showed good antioxidant and anti-glycosylation activities. The inhibition effects of CHP samples on AGE formation reached the highest of 37.5 ± 0.31% (0.5 mg/mL), 45.3 ± 0.29% (1.0 mg/mL), and 66.0 ± 0.35% (1.5 mg/mL), respectively, at 21 days of incubation. The molecular docking result demonstrated that the GalA of CHP interacted with the amino acid residues to form hydrogen bonds and hydrophobic groups and reduced AGE formation. This study provided some insights into the pectin polysaccharides of Arabica coffee husks, which could be applied to the food industry in the future. The future study will be focused on evaluating the emulsifying properties of CHP during beverage processing and its bioactivities through animal experiments.

## Figures and Tables

**Table 1:** Physicochemical characterization of CHP.

Parameter	Values
Purified yield (%)	19.13 ± 0.85
Moisture (%)	5.08 ± 0.23
Ash (%)	4.81 ± 0.33
Carbohydrate (%)	85.58 ± 1.02
Flavone (mg/kg)	1.39 ± 0.13
Polyphenol (mg/kg)	6.93 ± 0.10
DE (%)	38.81 ± 0.96
DA (%)	16.36 ± 0.75
Molecular weight	
Mn (g/mol)	4.24 × 10^5^
Mp (g/mol)	3.52 × 10^5^
Mw (g/mol)	1.04 × 10^6^
Mz (g/mol)	7.82 × 10^6^
Mw/Mn	2.46
Mz/Mw	7.52
Monosaccharide composition (%)	
Rha	2.55
GalA	45.01
GlcNAc	5.17
Glc	32.29
Gal	6.80
Xyl	0.76
Ara	7.42
Molar ratio	
HG (%)	42.46
RG I (%)	19.32
R_1_	2.68
R_2_	0.06
R_3_	5.58

HG (homogalacturonan) = galacturonic acid − rhamnose; RG I (rhamnogalacturonan I) = 2 rhamnose + arabinose + galactose; R_1_ = galacturonic acid/(rhamnose + arabinose + galactose); R_2_ = rhamnose/galacturonic acid; R_3_ = (Arabinose + Galactose) /Rhamnose. DE, degree of esterification; DA, degree of acetylation; Rha, rhamnose; GalA, galacturonic acid; GlcNAc, β-N-acetylglucosamine; Glc, glucose; Gal, galactose; Xyl, xylose; Ara, arabinose. Mn, number of average molecular weights; Mp, the molecular weight of the peak. Mw, average molecular weight; Mz, higher average molecular weight; Mw/Mn, polydispersity index; Mz/Mw, dispersion of molecular weight.

## Data Availability

Data are contained within the article and the data presented in this study are available upon request from the corresponding author.
